# Reviews via Mobile: The Role of Mobile Cues and Typographical Errors in Online Review Adoption

**DOI:** 10.3389/fpsyg.2022.861848

**Published:** 2022-04-15

**Authors:** Young-shin Lim, Ewa Maslowska

**Affiliations:** ^1^Graduate School of Business, Sejong University, Seoul, South Korea; ^2^Department of Advertising, University of Illinois at Urbana-Champaign, Urbana, IL, United States

**Keywords:** online reviews, mobile device, typographical error, effort, information usefulness, attitude, fundamental attribution error, information adoption model (IAM)

## Abstract

Online consumer reviews are increasingly being written on and posted from mobile devices such that some platforms have started to indicate when this is the case with cues such as “via mobile” (i.e., mobile cue). Reviews from mobile devices differ from those from non-mobile devices; for example, reviews from mobile devices are more likely to include typographical errors. For this study, a web-based experiment was conducted to investigate viewers’ evaluation and adoption of online reviews in regard to a mobile cue and typographical errors. The results indicate an interaction effect between the presence of a mobile cue and typographical errors. When a review did not include typographical errors, the presence of a mobile cue negatively affected the evaluation and adoption of information (i.e., the viewer’s attitude toward the reviewed restaurant). However, the effects of a mobile cue were not significant for a review with typographical errors. Further, the results suggest that the viewer’s perception of the review writing effort and the review’s information usefulness are sequential mediators explaining the information adoption mechanism. The findings provide interesting insights into consumers’ perceptions of online reviews in the current media landscape in which the large-scale adoption of mobile devices is a well-recognized phenomenon.

## Introduction

According to the Pew Research Center, as of early 2021, 85% of American adults owned a smartphone and 77% owned a desktop or laptop computer (2021). Mobile devices enable people to access the Internet from almost any location and to send and receive messages. As a result, a significant amount of user-generated content posted online at the present time is posted from mobile devices, including online consumer reviews, which have become an integral aspect of consumer decision making (Mariani et al., 2019; Podium, 2020). With the growth of online reviews written on and posted from mobile devices, platforms such as Tripadvisor.com indicate when a review was submitted from a mobile device, with a cue such as “via mobile” (i.e., mobile cue). However, the question as to how and to what extent mobile cues influence viewers’ perceptions of a review has been investigated in only a few research studies. To address this gap in the literature, we focus on the effects of mobile cues on information adoption in relation to typographical errors, which are more common in typed text on mobile devices than typed text on other devices (Dhakal et al., 2018; Palin et al., 2019). Specifically, we examine (1) the interaction between a mobile cue and typographical errors based on fundamental attribution error and (2) sequential mediation of information adoption (i.e., attitude formation based on the review) through viewers’ perceptions of review writing effort and information usefulness based on the effort heuristics and the information adoption model (IAM) (Sussman and Siegal, 2003; Kruger et al., 2004).

## Review of Literature and Hypotheses

### Mobile Cues

Mobile cues indicate that a text was written on and/or posted from a mobile device such as “sent from my iPhone,” “via mobile” (e.g., on Tripadvisor.com), and “Submitted from a mobile device” (e.g., on Booking.com) (Carr and Stefaniak, 2012; Grewal and Stephen, 2019). A mobile cue may function as a peripheral cue that viewers use in evaluating the quality of the information presented (Ransbotham et al., 2019). Mobile cues function in this peripheral way especially when viewers are processing information heuristically, which is particularly likely when confronting information overload—a commonplace phenomenon during online review browsing (Forman et al., 2008; Erkan and Evans, 2016).

How a mobile cue affects evaluations of an online consumer review depends on the associations that viewers have developed in respect to information posted from mobile devices (Ransbotham et al., 2019). One potential source of the associations is differences between mobile and non-mobile reviews. Researchers have indicated that depending on whether they are posted from a mobile or non-mobile device, reviews differ in terms of textual features such as length, valence, and word use (März et al., 2017; Jia and Wu, 2019; Ransbotham et al., 2019). Although there are some inconsistencies across the findings presented in previous studies depending on the platforms and the study periods, one consistent finding across several studies is that mobile reviews as compared with non-mobile reviews are shorter in length (Piccoli and Ott, 2014; Jia and Wu, 2019; Mariani et al., 2019; Zhu et al., 2020). Further, Zhu et al. (2020) reported that given their shorter length, in comparison with non-mobile reviews, mobile reviews are less likely to include topics such as price, product functionality, service quality, and logistics quality. Such results may be due to reviewers’ balancing of the overall cognitive cost of posting a review—i.e., less effort is expended in generating content in order to cancel out the relatively greater effort expended in inputting text on mobile devices (Zhu et al., 2020). Overall, it is reasonable to consider mobile review texts to be of lower quality than non-mobile review texts.

In a small number of studies, researchers have investigated consumers’ evaluations of mobile reviews vs. non-mobile reviews based on the number of helpful votes or likes associated with each kind of review. In a study by Mariani et al. (2019), mobile reviews received fewer helpful votes than did desktop reviews. Ransbotham et al. (2019) found that consumers rated mobile reviews as less valuable than non-mobile reviews based on the difference in the number of likes received. Their results suggested that in addition to the differences in mobile and non-mobile reviews’ content, the kind of device used to post a review has a direct effect on evaluations of the review. Further, the researchers explained the negative evaluations of mobile reviews as a consequence of learning the quality of mobile reviews. Initially, consumers may have valued mobile reviews for their novelty. However, over time with further experience reading mobile reviews, they may have started to devalue them. Thus, we can expect a mobile cue to negatively affect evaluations of a review thereby reducing its influence on consumers’ attitudes toward the product.

### Typographical Errors

One problem with texts from a mobile device is that they exhibit more typographical errors—so-called fat-finger errors—than is the case for texts from a non-mobile device. According to two large-scale analyses, people leave more errors when typing on mobile keyboards than when typing on desktop keyboards (2.34 and 1.17%, respectively) (Dhakal et al., 2018; Palin et al., 2019). Typing on mobile touchscreen keyboards, people are prone to make errors due to the small size of the keys and the absence of physical keys because without a physical reference point, visual attention is divided between the text display and the keyboard (Hoggan et al., 2008; Jiang et al., 2020). As a result, it is more difficult to detect errors and more cumbersome to correct them on a mobile device than on a non-mobile device (Jiang et al., 2020). Furthermore, contextual factors such as typing while moving and the surrounding physical environments may cause more typographical errors (Lurie et al., 2018). In sum, mobile reviews are more likely than non-mobile reviews to include errors of a typographical nature.

Typographical errors refer to mechanical errors that result from mistyping, such as striking a wrong key and switching the order of adjacent letters in a word (e.g., “wsa” instead of “was”) (Min et al., 2000). Typographical errors are distinguished from orthographical errors, which involve cognitive processing, such as phonetic misspelling (e.g., “hite” instead of “height”) (Min et al., 2000; Cox et al., 2017). Past studies have demonstrated that textual errors negatively influence evaluations of the communicator/communication in various contexts, including online reviews and e-mail communication (Lea and Spears, 1992; Vignovic and Thompson, 2010; Carr and Stefaniak, 2012; Schindler and Bickart, 2012; Cox et al., 2017; Cooper et al., 2020). Specifically, typographical errors are more likely to be attributed to “carelessness and clumsy or hurried typing,” whereas orthographical errors are more likely to be attributed to ignorance or a cognitive challenge (Boland and Queen, 2016, p. 3; Cox et al., 2017).

### Interaction of Mobile Cues and Typographical Errors

In addition to the individual effects of mobile cues and typographical errors on evaluations of online reviews, mobile cues may alter the inferences viewers make about online reviews with typographical errors by providing contextual information. When people encounter a communication problem such as a typographical error, they may attribute it either to dispositional factors (e.g., the sender’s carelessness, personality) or to situational factors (e.g., technology problems or other external constraints) (Vignovic and Thompson, 2010). People tend to attribute others’ behaviors to dispositional factors rather than to situational factors (i.e., fundamental attribution error or overattribution effect) (Tetlock, 1985; Vignovic and Thompson, 2010), and such attribution is more likely to occur in computer-mediated communication contexts where limited social cues exist (Lea and Spears, 1992; Cramton, 2001; Vignovic and Thompson, 2010). For example, Vignovic and Thompson (2010) study on cross-cultural email collaboration demonstrated that cultural cues suggesting the communicator is from a culture that differs from that of the receiver reduce negative perceptions due to spelling and grammatical errors. In terms of mobile cues, Carr and Stefaniak (2012) demonstrated that the presence of a cue of this kind (i.e., “Sent from my iPhone”) can mitigate the negative effect of textual errors (i.e., grammatical errors) on evaluations of the email sender’s professionalism. In a similar vein, when a review presents a cue indicating that it was written on/posted from a mobile device, based on their knowledge that mobile typing is more prone to typographical errors and requires more effort to fix the errors, viewers may attribute typographical errors to the situation (i.e., mobile typing) rather than to the reviewer (Jiang et al., 2020). Such a mitigation effect is likely to exist in the context of online reviews as well.

### Perceived Review Writing Effort and Information Usefulness as Mediators in Information Adoption

According to recent surveys, many consumers consult online consumer reviews before making purchases and make decisions based on those reviews (Podium, 2020; ReviewTrackers, 2021). On the other hand, people selectively adopt information due to the overflow of online reviews and other electronic word of mouth (eWOM) with varying degrees of information quality and credibility (Erkan and Evans, 2016). In consideration of the current media landscape, in the present study, we examine the perceived review writing effort and information usefulness as sequential mediators in the mechanism through which mobile cues and typographical errors affect online review adoption.

Effort refers to the level of mental or physical activity invested in meeting a goal (Inzlicht et al., 2018). According to the effort heuristic, people often judge quality based on a perception of effort because it is difficult to determine the quality of a product, whereas it is easier to assess the effort invested (Kruger et al., 2004). Kruger et al. (2004) argued that “effort is a generally reliable indicator of quality” and “people use effort as a heuristic for quality” (p. 92). In this sense, the perceived effort entailed in writing and posting a piece of information may function as a proxy for information quality, thereby affecting information usefulness and the viewer’s adoption of information.

In review writing, perceived effort concerns the resources (e.g., time, cognitive energy) that the reviewer put into writing and posting a review (Grewal and Stephen, 2019). Grewal and Stephen (2019) demonstrated that perceived review writing effort mediates the effects of device type on purchase intention. Whereas the previous study has highlighted the point that writing on mobile devices takes more physical effort due to the physical properties of the devices (e.g., a small screen and keyboard) (Grewal and Stephen, 2019), review writing effort involves more factors such as writing situation (e.g., outdoors) and cognitive effort in generating thoughts. As mobile writing has become widespread, review readers are more likely to focus on the decreased cognitive effort in generating review content due to typing on mobile devices, rather than appreciating the mobile typing effort. Thus, readers’ experiences with mobile reviews that are shorter and include fewer content dimensions than non-mobile reviews are likely to lead to associating mobile reviews with less effort as compared to the effort associated with non-mobile reviews (Mariani et al., 2019; Zhu et al., 2020). We, therefore, consider the effect of mobile cues on the perceived review writing effort and propose the following hypothesis:

H1: Viewers will perceive an online review to be written with less effort when a mobile cue is present.

Further, typographical errors may function as a signal of low effort. According to literature in computer-mediated communication, viewers relate typographical or grammatical errors to some quality of the sender such as carelessness, hurriedness, and/or a lack of conscientiousness (Lea and Spears, 1992; Vignovic and Thompson, 2010). Similarly, viewers are likely to devalue the reviewer’s effort when a review includes typographical errors. In this regard, we propose the following hypothesis:

H2: Viewers will perceive an online review to be written with less effort when typographical errors are present.

Moreover, the presence of typographical errors is expected to change the effect of a mobile cue on the perceived review writing effort. As noted, providing situational information that the review was written on and/or posted from a mobile device may mitigate the fundamental attribution error because people are familiar with the fact that typing on a mobile keyboard tends to produce more typographical errors and requires more time and effort to correct the errors than on non-mobile devices (Dhakal et al., 2018; Palin et al., 2019; Jiang et al., 2020). Thus, when a review includes typographical errors, a mobile cue may have a positive effect on the perceived review writing effort by mitigating the effect of typographical errors and so canceling out or even exceeding the negative effect of the mobile cue by itself. Thus, we propose the following hypothesis:

H3: There will be an interaction effect between mobile cue and typographical errors on perceived review writing effort such that a mobile cue negatively affects the perceived review writing effort for a review without typographical errors, but not for a review with typographical errors.

Information usefulness refers to “people’s perception that using new information will enhance [their] performance” (Erkan and Evans, 2016, p. 50). The information adoption model (IAM) posits information usefulness as a pivotal construct in informational influence processes, i.e., as affecting the degree of influence (Eagly and Chaiken, 1993; Sussman and Siegal, 2003). Specifically, in the IAM, information usefulness mediates the effects of argument quality and source credibility—corresponding to central and peripheral processes of persuasion—on information adoption. Extending the IAM, the information acceptance model (IACM) proposes information usefulness as a mediator in eWOM adoption. Studies have demonstrated the mediating role of information usefulness in linking the effects of review/reviewer factors such as information quality and review credibility on information adoption (Cheung et al., 2008; Cheung, 2014; Erkan and Evans, 2016). Similarly, Kim et al. (2018) demonstrated the effect of review helpfulness votes on e-commerce purchase probability.

According to the effort heuristic, perceived review writing effort can function as a heuristic for information quality—an antecedent of information usefulness. Hence, the perceived review writing effort induced by a mobile cue and typographical errors may affect the information usefulness of the review. Thus, we propose the following hypothesis:

H4: The interaction effect of mobile cue and typographical errors on information usefulness will be mediated by the perceived review writing effort.

Furthermore, following the IAM and the IACM, induced information usefulness may affect the extent of a review’s informational influence. Thus, we propose the following hypothesis:

H5: The interaction effect of a mobile cue and typographical errors on attitude toward the reviewed object will be serially mediated by the perceived review writing effort and the information usefulness of the review.

## Materials and Methods

### Study Design and Participants

A 2 (mobile cue: absent vs. present) × 2 (typographical errors: absent vs. present) online experiment was conducted to test the hypotheses. A total of 178 participants (64.0% females, *M*_*Age*_ = 43.16, *SD*_*Age*_ = 14.06) were recruited from TurkPrime US panels. Participant recruitment through crowdsourcing is widely used in consumer research and other social science disciplines (Goodman and Paolacci, 2017; Litman et al., 2017). In particular, we used TurkPrime US panels to recruit English-speaking internet users and recruited workers from 18 to 65 of age. After indicating their agreement to the conditions of participation on the consent form provided, the participants were introduced to a hypothetical situation according to which they were interested in going to an Italian restaurant in town and were checking online reviews for the restaurant. Next, the participants were randomly assigned to one of the conditions and exposed to a fictitious restaurant review on the following page. The conditions were operationalized through the stimuli of presenting an online consumer review.

### Stimuli

**FIGURE 1:**
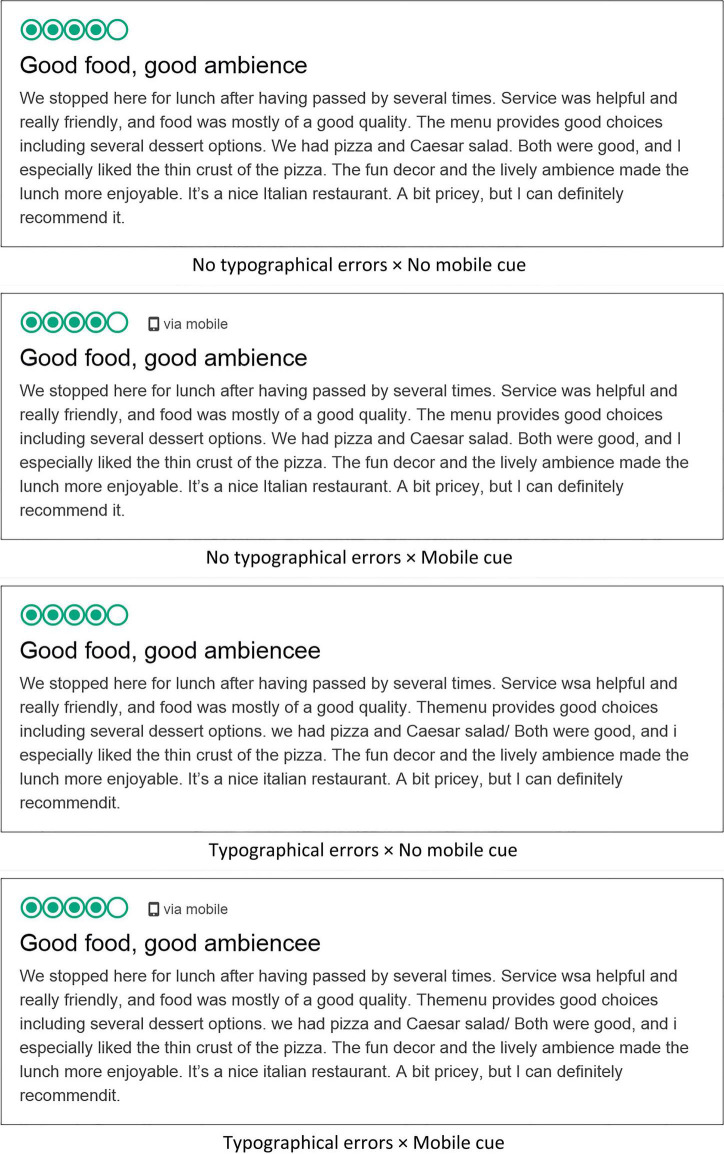
Stimuli.

The experimental stimuli were created based on the review format of Tripadvisor.com —the reviewer’s overall rating, the review title, and the review text. The top two lines presented the star-rating and the review title. Immediately below that, a moderately positive review with 79 words was presented. Figure 1 presents all four stimuli. The mobile cue variable was operationalized by the absence vs. presence of a smartphone icon and the text “via mobile” next to the star-rating on the first line. The typographical errors variable was operationalized by manipulating the review title and the main text. The typographical errors conditions displayed eight errors based on QWERTY keyboard layout, whereas the no errors condition did not include any errors.

### Measures

After seeing the review, the participants completed a questionnaire designed to measure their perceptions of the reviewer’s effort, their perceptions of the review’s information usefulness, their attitudes toward the reviewed restaurant, and their general attitudes toward online restaurant reviews.

#### Perceived Review Writing Effort

Perceptions of the effort invested by the reviewer in writing the review were measured with three Likert scale items (1 = strongly disagree vs. 7 = strongly agree) adapted from Grewal and Stephen (2019): “The reviewer put a lot of effort into writing this review,” “The reviewer took time to craft this review,” and “The reviewer put a lot of thought into this review” (Cronbach’s α = 0.92).

#### Information Usefulness

The participants were asked to indicate how well each of four adjectives—valuable, informative, helpful, and useful—described the review they had read (1 = describes very poorly vs. 7 = describes very well). The first three items (valuable, informative, and helpful) were adapted from Bailey and Pearson (1983) and Cheung et al. (2008). The reliability (Cronbach’s α) of the four items was 0.95.

#### Attitude Toward the Reviewed Restaurant

Attitude toward the reviewed restaurant was measured with five 7-point bipolar scale items: bad-good, negative-positive, unacceptable-acceptable, unfavorable-favorable, unpleasant-pleasant (first four items adapted from Burgoon et al., 1978). The reliability (Cronbach’s α) of the five items was 0.94.

#### General Attitude Toward Restaurant Reviews

In addition to the mediators and the dependent variable, general attitude toward online restaurant reviews (Park and Kim, 2008) was included as a covariate for all the analyses based on the IACM according to which consumer characteristics such as the need for information and attitude toward information affect information usefulness and further information adoption (Erkan and Evans, 2016). The participants were asked to indicate their level of agreement with three statements about restaurant reviews written by other consumers in general (1 = strongly disagree vs. 7 = strongly agree): “Before visiting restaurants, I read online consumer reviews,” “Online restaurant reviews by other consumers are helpful for my decision making,” and “Consumer-generated restaurant reviews make me confident in visiting restaurants” (Park and Kim, 2008). The reliability (Cronbach’s α) of the three items was 0.87.

## Results

Before we tested the hypotheses, the manipulation of the typographical errors variable was examined. The results indicated a significant effect of manipulation of the typographical errors, *F*_(1, 176)_ = 77.59, *p* < 0.001, partial η^2^ = 0.31, *M*_*no errors*_ = 5.58, *SD* = 1.28, and *M*_*typographical errors*_ = 3.40, *SD* = 1.98 (1 = poor typing skill to 7 = good typing skill) (Cox et al., 2017). Moreover, the zero-order correlations between the mediators, the dependent variable, and the covariate were checked. Table 1 provides the correlations and the means and standard deviations of the variables.

**TABLE 1:** Zero-order correlations, means, and standard deviations.

Variable	1	2	3	4	*M*	*SD*
1. Perceived review writing effort	1	0.67***	0.43***	0.19*	4.56	1.62
2. Information usefulness of the review		1	0.57***	0.23**	5.42	1.19
3. Attitude toward the reviewed restaurant			1	0.31***	5.81	1.07
4. General attitude toward online restaurant reviews				1	4.92	1.50

*p < 0.05.

**p < 0.01.

***p < 0.001.

A 2-way ANOVA was conducted to test the interaction between mobile cue and typographical errors and the two main effects (H1, H2, and H3).

For Hypothesis 1, we examined the main effect of a mobile cue. According to the 2-way ANOVA, the main effect of the mobile cue was not significant: *F*_(1, 173)_ = 0.40, *p* = 0.529, partial η^2^ = 0.002. The viewers’ perception of review writing effort did not differ between the participants who were exposed to a review with a mobile cue (*M* = 4.43, *SD* = 1.64) and the participants who were exposed to a review without a mobile cue (*M* = 4.69, *SD* = 1.61).

For Hypothesis 2, we examined the main effect of typographical errors. The 2-way ANOVA revealed a significant main effect of typographical errors: *F*_(1, 173)_ = 30.88, *p* < 0.001, partial η^2^ = 0.151. The participants who were exposed to a review with typographical errors perceived the reviewer as having expended less effort in writing the review (*M* = 3.91, *SD* = 1.66) than did the participants who were exposed to a review without typographical errors (*M* = 5.15, *SD* = 1.35).

**FIGURE 2:**
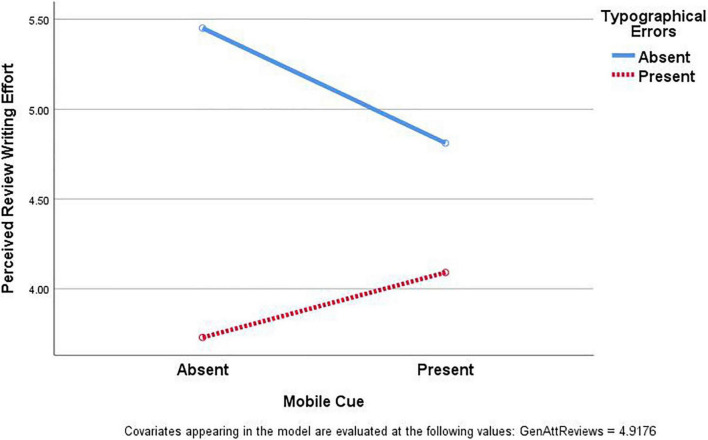
Interaction between mobile cue and typographical errors on perceived review writing effort.

For Hypothesis 3, we examined the interaction between mobile cue and typographical errors on perceived review writing effort. The two-way ANOVA revealed a significant Mobile × Typo interaction on perceived review writing effort: *F*_(1, 173)_ = 5.14, *p* = 0.025, partial η^2^ = 0.029. Figure 2 illustrates the interaction pattern. Two sets of one-way ANOVAs were conducted to analyze the interaction pattern. For a review without typographical errors, an ANOVA revealed a negative effect of mobile cue on perceived review writing effort: *F*_(1, 90)_ = 5.86, *p* = 0.017, partial η^2^ = 0.061. Without typographical errors, a review was perceived as having been written with less effort when presented with a mobile cue (*M* = 4.83, *SD* = 1.43) than when presented without a mobile cue (*M* = 5.45, *SD* = 1.22). However, for a review with typographical errors, the difference between a review presented with a mobile cue (*M* = 4.04, *SD* = 1.75) and a review without a mobile cue (*M* = 3.77, *SD* = 1.55) was not significant: *F*_(1, 82)_ = 0.90, *p* = 0.346, partial η^2^ = 0.011.

For Hypotheses 4 and 5, we examined the mediating roles of perceived review writing effort and the information usefulness of the review in the relationship between mobile cue, typographical errors, and the viewer’s attitude toward the reviewed restaurant. A set of mediation analyses was conducted using SPSS macro PROCESS version 4.0 with 10,000 bootstrap samples (Hayes, 2022).

For Hypothesis 4, we examined whether perceived review writing effort mediates the effect of the Mobile × Typo interaction on information usefulness (Mobile × Typo → Perceived effort → Information usefulness). The moderated mediation was tested with Model 8 of PROCESS. The results indicate a significant moderated mediation path whereby the Mobile × Typo interaction affects the perceived usefulness of the review through the perceived effort of the reviewer: point estimate = 0.46 (*SE* = 0.21), 95% bootstrap CI = [0.0541, 0.8716]. Specifically, perceived review writing effort mediated the effect of the mobile cue on information usefulness when the review did not include any typographical errors: point estimate = −0.29 (*SE* = 0.13), 95% bootstrap CI = [−0.5523, −0.0502]. However, the mediation was not significant when the review did include some typographical errors: point estimate = 0.17 (*SE* = 0.16), 95% bootstrap CI = [−0.1624, 0.4863]. The direct effects of the mobile cue, the typographical errors, and the interaction on information usefulness were not significant in the model.

**FIGURE 3:**
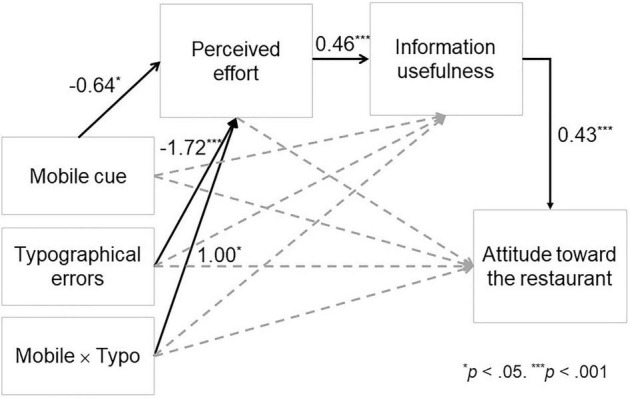
Summary of the direct paths. The solid arrows indicate the significant direct paths, and the numbers next to the arrows are the path coefficients.

For Hypothesis 5, we examined the serial mediation effect of the Mobile × Typo interaction on the viewer’s attitude toward the reviewed restaurant through perceived review writing effort and the information usefulness of the review (Mobile × Typo → Perceived effort → Information usefulness → Attitude). The moderated serial mediation was tested with Model 85 of PROCESS. The data supported a significant moderated serial mediation: point estimate = 0.20 (*SE* = 0.11), 95% bootstrap CI = [0.0181, 0.4682]. For a review that did not include any typographical errors, the mobile cue negatively affected attitude through perceived review writing effort and information usefulness: point estimate = −0.13 (*SE* = 0.07), 95% bootstrap CI = [−0.2942, −0.0176]. However, the serial mediation was not significant when the review did include some typographical errors: point estimate = 0.07 (*SE* = 0.08), 95% bootstrap CI = [−0.0734, 0.2459]. The direct effects of the typographical errors, the mobile device cue, and the interaction on the viewer’s attitude toward the restaurant were not significant in the model. Figure 3 presents a summary of the direct paths.

## Discussion

Online consumer reviews have become an essential source of information for consumers. Reviews are increasingly read and posted via mobile devices (Mariani et al., 2019; BrightLocal, 2020), and some platforms inform viewers when reviews are submitted from mobile devices. However, research focused on how mobile cues affect viewers’ evaluations of the reviews and adoption of information remains limited. In this regard, we investigated the effect of mobile cues in relation to typographical errors—a commonplace feature of reviews written on/posted from mobile devices.

Our primary findings suggest that the effects of mobile cues and typographical errors on perceived review writing effort interact (H3 supported). Individually, both mobile cues and typographical errors functioned as cues signaling the expenditure of only limited review writing effort. However, working in combination, the presence of a mobile cue mitigated the negative effect of typographical errors and canceled out its negative effect. The interaction qualified the main effect of the typographical errors (H2 supported) but not the main effect of the mobile cue (H1 not supported). Further, our results show that the interaction between a mobile cue and typographical errors affects consumers’ attitudes toward the product or service reviewed (a restaurant in this case) and that this effect is sequentially mediated by perceived review writing effort and information usefulness (H4 and H5 supported). On reading a review without typographical errors, the participants perceived greater review writing effort in the absence as compared to the presence of a mobile cue. Further, this greater perceived effort led to a higher level of information usefulness and more favorable attitudes toward the product/service reviewed. Serial mediation between a mobile cue and attitude toward the product/service was not significant for a review with typographical errors.

First, for a review without typographical errors, a mobile cue negatively affected the perception of review writing effort, and the effect was mediated to information usefulness and attitude toward the product. These results are in line with studies suggesting that mobile reviews receive less favorable evaluations than do non-mobile reviews (Mariani et al., 2019; Ransbotham et al., 2019). Consumers are likely to build associations in respect to mobile writing through reading mobile reviews or other texts posted from mobile devices (Ransbotham et al., 2019). Being exposed to mobile reviews that are shorter than non-mobile reviews and deficient in content by comparison (Mariani et al., 2019; Zhu et al., 2020), consumers would have started to associate mobile reviews with less effort and lower quality. Moreover, consumers’ own experiences with mobile writing could have affected perceptions of mobile reviews as well. Mobile writing can take place in a broad range of physical environments, and it is often the case that people have limited mental resources to focus on writing (Lurie et al., 2018; Schröder et al., 2019). Such experiences can lead consumers to think of mobile reviews as involving less effort than those written from a desktop.

On the other hand, the negative effects of mobile cues are contrary to Grewal and Stephen (2019) findings, according to which a mobile cue induced perceptions of greater review writing effort because people consider writing on a mobile device to be more physically effortful due to the physical properties of the devices (e.g., a small screen and keyboard). One potential explanation for the inconsistency between the findings from Grewal and Stephen (2019) and those of the present study is the difference in the study periods. In the early days of smartphone dissemination, people would have been less familiar with mobile typing and more likely to appreciate others’ physical effort in typing on mobile devices. However, as people have become used to mobile typing, they are less likely to put emphasis on the physical effort involved and more likely to recognize the division of mental resources accompanied in many mobile writing situations. In this regard, the findings of the present study suggest a changing conceptualization of mobile writing.

Second, typographical errors negatively affected the perception of review writing effort. The result extends the literature in computer-mediated communication that demonstrated negative effects of textual errors on the senders’ impression (Lea and Spears, 1992; Vignovic and Thompson, 2010; Carr and Stefaniak, 2012) and suggests perceived review writing effort as another variable that explains the mechanism through which textual errors affect the adoption of online consumer review. In particular, typographical errors result from mistyping rather than ignorance or a cognitive challenge (Min et al., 2000; Boland and Queen, 2016; Cox et al., 2017). Thus, to the viewers, the fact that a reviewer submitted the review without correcting the errors can mean that the reviewer did not put enough effort into writing the review.

Third, there was a significant interaction between the mobile cue and typographical errors. The effects of the mobile cue differed depending on whether or not the review included typographical errors. For a review without typographical errors, the mobile cue led to a perception of limited review writing effort. However, for a review with typographical errors, the perception of effort did not differ in relation to whether or not a mobile cue was present. These results suggest that whereas a mobile cue may suggest less effort invested by the reviewer, it also provides some justification for typographical errors. Hence, the negative effects of a review being written on and posted from a mobile device and the positive effects of justifying typographical errors cancel each other out for a review with typographical errors. Moreover, although the difference was not significant, for a review with typographical errors, the perceived effort was greater when a mobile cue was present (Figure 2). For the present study, the typographical errors conditions presented eight errors in a relatively short review. Thus, the mobile cue might not have been sufficient to justify the number of errors presented. It may be that the presence of a mobile cue would have had a greater mitigating influence for a review with fewer typographical errors.

Further, the interaction results are consistent with previous findings that demonstrated how mobile cues and other cues that provide situational information could mitigate negative perceptions arising from textual errors (Lea and Spears, 1992; Cramton, 2001; Vignovic and Thompson, 2010; Carr and Stefaniak, 2012). Our findings extend the literature on fundamental attribution error and computer-mediated communication by demonstrating the role of mobile cues in reducing fundamental attribution errors in online consumer review contexts. In addition to the theoretical contributions, our findings have implications for the design of online review communities and other community platforms where information is exchanged among strangers. Many platforms support viewers’ understanding of communication by providing information about the communicators via short profiles. Yet, information about the device used is neglected on many platforms. Given the influence of submission devices on the textual features of a review and the wide adoption of smartphones, providing device information could help viewers in terms of using online reviews (März et al., 2017; Jia and Wu, 2019; Ransbotham et al., 2019).

Fourth, the results of the present study demonstrate a sequential mediation path from the mobile cue and typographical errors to attitude toward the reviewed product. Our data support perceived review writing effort and information usefulness as the sequential mediators. The concept of information usefulness has been highlighted as a critical mediator in informational influence and information adoption (Eagly and Chaiken, 1993; Sussman and Siegal, 2003). In particular, the concept has received considerable attention in the online consumer review context, where anyone can share information and consumers are bombarded with information of extremely varied quality (Erkan and Evans, 2016). The results of the present study support the mediating role of information usefulness and the relevance and efficacy of both the IAM and IACM.

Moreover, the results indicate that perceived review writing effort functions as a mediator that links the effects of mobile cues and typographical errors to information usefulness. The effort heuristic suggests effort as a heuristic for quality (Kruger et al., 2004). Grewal and Stephen (2019) proposed perceived review writing effort as a mediator that determines the adoption of mobile reviews. Our results support the literature and extend it by demonstrating that perceived review writing effort mediates the effect of typographical errors as well. We expect perceived review writing effort to become a more important construct in the adoption of information from social media in this age of ubiquitous computing and multitasking.

Speaking of limitations, differences in people’s experiences with mobile writing and changes in technology may undermine to some extent the generalizability of the present study. The heuristic effects of a mobile cue by itself and in combination with typographical errors come from people’s experiences with mobile writing (Ransbotham et al., 2019). However, the experiences vary given the existence of the mobile digital divide and generation gaps in the adoption and use of mobile devices (Dempsey and Sun, 2020; Pew Research Center, 2021). Specifically, Palin et al. (2019) reported a generation gap in mobile typing speed. Such differences in experience may influence people’s conceptualization of mobile tasks, and interpretations and evaluations of mobile reviews are likely to differ across age groups. Future research examining how different age groups respond to mobile cues would broaden our understanding of the influence of mobile cues and other kinds of cues likewise. Further, innovations in mobile input methods and technology that aids mobile typing (e.g., autocorrection) can also alter people’s ideas of mobile typing as well as the relationship between mobile typing and typographical errors. In this sense, the generalizability of the findings may change with advances in technology.

In addition, cultural differences may be a factor in the nature and extent of the effects of typographical errors. The present study was designed in the context of online reviews written in English using the QWERTY layout keyboard, and the experiment was conducted with US panels. However, there are various writing systems, and not all use phonetic alphabets. The influence of typographical errors on how a text is understood could vary across languages, and it may be that cultures differ in terms of attitudes toward typographical errors. In a similar sense, intentional misspellings are prevalent in social media (Agarwal and Yiliyasi, 2010). People familiar with such a culture might also respond differently to typographical errors. In this regard, linguistic characteristics and cultures are likely to function as boundary conditions for the findings such that future investigations are needed in this area.

In conclusion, the results suggest that mobile cues and typographical errors affect the adoption of online review information. Further, the results suggest that perceived review writing effort and review usefulness function as sequential mediators explaining the mechanism. In particular, the findings indicate that mobile cues can influence people’s processing of the textual characteristics of online reviews and further affect information adoption. Overall, the results provide important insights into consumers’ perceptions of online reviews in the current media landscape, where reviews are increasingly posted from and read via mobile devices.

## Data Availability Statement

The raw data supporting the conclusions of this article will be made available by the authors, without undue reservation.

## Ethics Statement

The studies involving human participants were reviewed and approved by the Amsterdam School of Communication Research, University of Amsterdam. The Ethics Committee waived the requirement of written informed consent for participation.

## Author Contributions

YL and EM contributed to the conception of the study and data collection. YL analyzed the data and reported the results in consultation with EM. Both authors contributed to manuscript writing and approved the submitted version.

## Conflict of Interest

The authors declare that the research was conducted in the absence of any commercial or financial relationships that could be construed as a potential conflict of interest.

## Publisher’s Note

All claims expressed in this article are solely those of the authors and do not necessarily represent those of their affiliated organizations, or those of the publisher, the editors and the reviewers. Any product that may be evaluated in this article, or claim that may be made by its manufacturer, is not guaranteed or endorsed by the publisher.
